# Antioxidant Properties of *Platycladus orientalis* Flavonoids for Treating UV-Induced Damage in Androgenetic Alopecia Hair

**DOI:** 10.3390/molecules29122876

**Published:** 2024-06-17

**Authors:** Chuntao Xu, Jiawei Dai, Weian Du, Hongbing Ji

**Affiliations:** 1 School of Chemistry and Chemical Engineering, Guangxi University, Nanning 530004, China; chuntao@zspt.edu.cn (C.X.); 2114402022@st.gxu.edu.cn (J.D.); 2 School of Information Engineering, Zhongshan Polytechnic, Zhongshan 528400, China; 3 Guangdong Homy Genetics Ltd., Foshan 528000, China; duwa168@163.com; 4 State Key Laboratory Breeding Base of Green-Chemical Synthesis Technology, Institute of Green Petroleum Processing and Light Hydrocarbon, College of Chemical Engineering, Zhejiang University of Technology, Hangzhou 310014, China

**Keywords:** *Platycladus orientalis* flavonoids, hair protection, ultraviolet, hair melanin

## Abstract

Background: Androgenetic alopecia (AGA) causes thinning hair, but poor hair quality in balding areas and damage from UV radiation have been overlooked. Plant extracts like *Platycladus orientalis* flavonoids (POFs) may improve hair quality in AGA. This study examines POFs’ effectiveness in treating AGA-affected hair and repairing UV-induced damage. Methods: Hair samples were analyzed using scanning electron microscopy (SEM) to examine surface characteristics, electron paramagnetic resonance (EPR) spectroscopy to measure free radicals in the hair, and spectrophotometry to assess changes in hair properties. Results: POFs effectively removed hydroxyl radicals from keratinocytes and had antioxidant properties. They also reduced UV-induced damage to AGA hair by mitigating the production of melanin free radicals. Following POF treatment, the reduction in peroxidized lipid loss in AGA hair was notable at 59.72%, thereby effectively delaying the progression of hair color change. Moreover, protein loss decreased by 191.1 μ/g and tryptophan loss by 15.03%, ultimately enhancing hair’s tensile strength. Conclusion: compared to healthy hair, hair damaged by AGA shows more pronounced signs of damage when exposed to UV radiation. POFs help protect balding hair by reducing oxidative damage and slowing down melanin degradation.

## 1. Introduction

*Platycladus orientalis* (L.) leaves, listed in the 2015 Chinese Pharmacopoeia for their bitter and cold properties [1], have been the subject of chemical composition studies revealing the presence of flavonoids [2], volatile oils [3], tannins, and beneficial inorganic elements [4]. Contemporary pharmacological research has demonstrated various potential therapeutic effects of *Platycladus orientalis* leaves, including anti-inflammatory, cough-suppressant, expectorant, hair-darkening, anti-tumor, antibacterial, cardiovascular, hemostatic, and neuroprotective properties [5]. Additional studies have investigated the potential promoting effects of volatile oils derived from *Platycladus orientalis* on hair growth [6]. While *Platycladus orientalis* flavonoids (POFs) have demonstrated antioxidant properties [7], limited research has been conducted on the potential therapeutic effects of POFs on androgenetic alopecia (AGA) hair, particularly in the context of repairing damage caused by UV exposure.

Hair is a high molecular weight keratin fiber that serves the dual purpose of protection for the scalp and aesthetics. While hair keratin fibers possess inherent inert properties, external factors such as light, heat, acidity, alkalinity, oxidants, and reducing agents can induce damage [8]. AGA is a prevalent form of patterned hair loss that impacts both genders. The miniaturization of hair follicles in balding regions in AGA renders hair more vulnerable to environmental harm [9]. Among the numerous factors contributing to hair damage, ultraviolet (UV) radiation emerges as a significant and modifiable risk factor [10,11]. The significance of hair in shaping personal image is paramount, with its psychological implications outweighing its physiological functions [12].

Consequently, individuals prioritize safeguarding their hair from UV damage. The assessment of hair health can be conducted through the evaluation of its visual appearance and structural integrity. Indicators such as tensile strength, ease of combing, color, shine, and electron microscopy [13] are utilized to assess the effectiveness of hair care products. The interior of hair conceals a range of structural material damages, such as hair proteins, amino acids, and lipid peroxidation [14], which serve as valuable evaluation indicators.

Hair can resist UV radiation primarily due to melanin, specifically eumelanin and pheomelanin. Melanin, a paramagnetic polymer containing many ortho semiquinone free radicals [15], facilitates drug interaction. Recent studies indicate that melanin can neutralize free radicals [16], particularly reactive oxygen species (ROS) produced by UV radiation [17]. However, prolonged exposure to UV rays can lead to the degradation of melanin, diminishing its protective properties on hair [18]. Currently, there is a lack of research examining the impact of superoxide free radicals on hair damage due to their fleeting nature, which makes them challenging to measure [19]. Nevertheless, it is feasible to utilize the endogenous free radicals in hair melanin to assess the effects of light exposure on hair damage. The objective of this study is to investigate this phenomenon.

The study aimed to investigate the potential protective properties of ingredients derived from the natural plant *Platycladus orientalis* against UV damage in AGA hair. The effectiveness of hair care was assessed using routine efficacy evaluation methods to offer new insights into indicators of hair structure damage.

## 2. Results

### 2.1. Analysis of Components of Extracts from Platycladus orientalis Leaves

*Platycladus orientalis* leaves contain multiple types of medicinally active ingredients, including flavonoids, volatile oils, tannins, polysaccharides, and inorganic salts. *Platycladus orientalis* leaf extracts have always been used as a traditional Chinese medicine in China. Some studies suggest that the volatile oils in *Platycladus orientalis* leaves have germinal effects, while POFs have anti-inflammatory and antioxidant effects. As shown in Table 1, GC -MS was used to analyze the components of flavonoids in POFs, which included eight flavonoids. They are, respectively, Myricetin, Quercetin, Afzelin, Aromadendrin, Hinokiflavone, Amentoflavone, Kaempferol, and Rutin. Among these flavonoid components, the contents of Myricetin, Hinokiflavone, and Amentoflavone were relatively high.

### 2.2. The Impact of UV Radiation on the Hair of Individuals with AGA and Healthy Individuals

**Figure 1:**
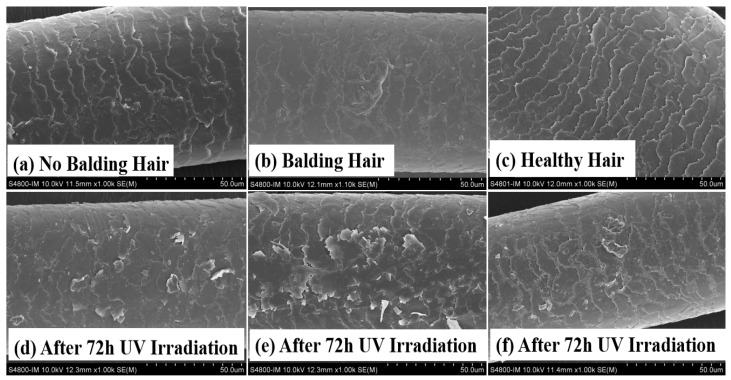
Comparison of SEM images depicting the effects of UV irradiation on frontal hair, posterior hair with AGA, and healthy individuals’ hair. Specifically, the images show (**a**) permanent hairs in the occipital region of individuals with AGA, (**b**) hairs in the balding area of individuals with AGA, (**c**) hairs in healthy individuals, (**d**) permanent hairs after 72 h of UV irradiation, (**e**) balding hairs of individuals with AGA after 72 h of UV irradiation, (**f**) healthy individuals’ hairs after 72 h of UV irradiation.

SEM was used to examine and compare the hair surface to assess the variances between AGA frontal hair and permanent hair on the posterior head. Hair specimens were obtained from 20 Chinese male AGA patients aged from 38 to 42 years. The SEM images in Figure 1b reveal minor damage to the frontal hair of the AGA patients, contrasting with the intact condition of permanent hair in Figure 1a. Following 72 h of UV irradiation, a notable deterioration of the frontal hair surface in AGA patients was evident, characterized by extensive scale curling. The findings depicted in Figure 1d,e suggest that the frontal hair of individuals with AGA exhibits greater vulnerability to UV damage than the permanent hair in the hindbrain. On the other hand, we found that the hair of healthy individuals had a better tolerance to ultraviolet rays, as can be seen from Figure 1c,f. After 72 h of UV irradiation, the degree of damage to healthy individuals’ hair was lower than the damage to the hair of individuals with AGA.

### 2.3. Hair’s Lipid Protection

**Figure 2:**
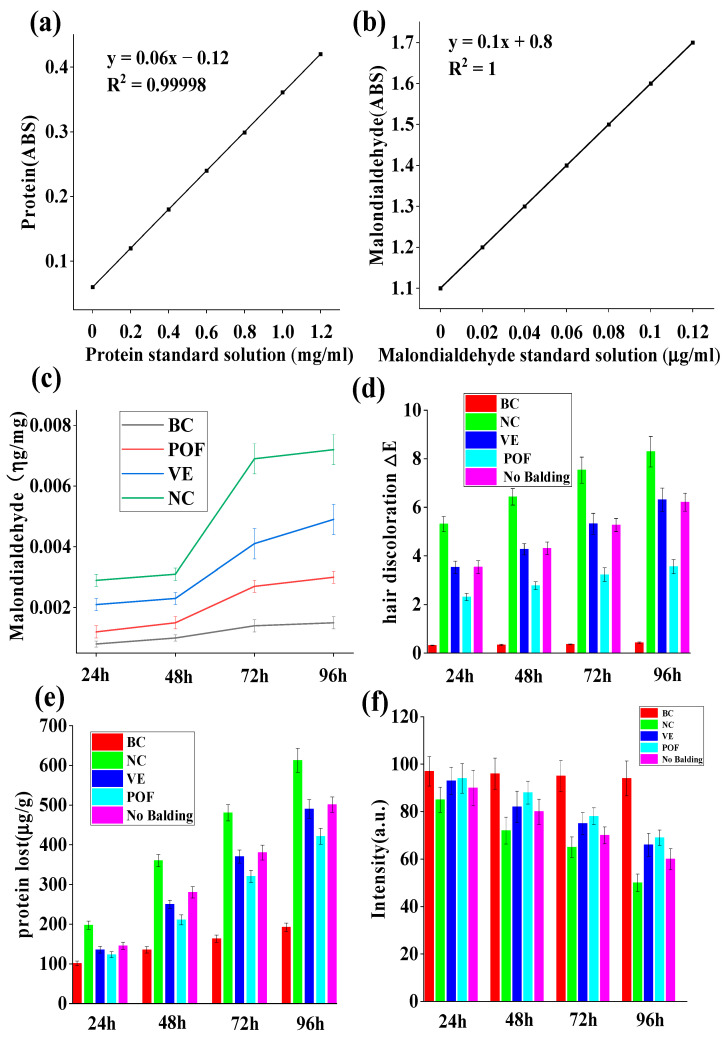
The effect of POF on lipid peroxidation, color, protein, and tryptophan loss in AGA hair under UV irradiation. (**a**) Standard curve for hair protein. (**b**) Standard curve for hair tryptophan. (**c**) POF protection of lipids in balding area hairs exposed to UV irradiation. (**d**) POF protection of hair color in balding areas under UV irradiation. (**e**) POF protection against hair protein loss in balding areas under UV irradiation. (**f**) POF protection against hair tryptophan loss in balding areas under UV irradiation. The experiments were performed in triplicate and the average value was taken.

The damage to lipids induced by UV irradiation is primarily attributed to the sequential generation of free radicals resulting from radiation, leading to lipid peroxidation [20]. UV irradiation promotes the production of free radicals, which have the same oxidation effect on unsaturated fatty acids in hair as ordinary oils, leading to a rancidity reaction. Malondialdehyde is the most typical product of lipid peroxidation [21]. Malondialdehyde reacts with thiosulfate barbiturate dye to form a pink compound with an absorption peak at a specific wavelength [22]. This property can be used to quantitatively analyze the content of malondialdehyde and calculate the degree of lipid peroxidation. The control group, which was not exposed to UV radiation, exhibited minimal lipid loss. Conversely, the NC group lacking protective measures demonstrated a lipid loss of 0.0072 ± 0.0005 μg/mg following 96 h of UV irradiation, a value nearly five times higher than that of the BC group, which had 0.0015 ± 0.0002 μg/mg. Figure 2c reveals a reduction in hair lipid loss by 0.0029 ± 0.0002 μg/mg post-POF treatment. This treatment resulted in a 59.72% decrease in hair lipid loss compared to the NC group. POF group has been shown to exhibit antioxidant properties, resulting in a deceleration of the oxidation and rancidity of hair lipids. Additionally, compared to hair loss areas, a reduced loss of lipids in non-hair loss areas of androgenetic AGA was observed.

### 2.4. Hair Discoloration

Two primary factors influence hair color: the degradation and fading of the natural hair pigment melanin particles and the yellowing of hair due to the decomposition and loss of tryptophan. The alteration in color is typically quantified by ΔE, which encompasses three color stimulus values: Hue, Value, and Chroma [23]. Figure 2d demonstrates that following 96 h of UV irradiation, minimal color difference was observed in the BC group, with ΔE = 0.43 ± 0.035. However, without protection, the NC group exposed to UV radiation exhibited notable color change (ΔE = 8.29 ± 0.631). Compared to the NC group, the POF group (ΔE = 3.56 ± 0.292) demonstrated a significant protective effect against hair fading and outperformed the VE group, with ΔE = 6.31 ± 0.48. Additionally, hair within the AGA hair loss region displayed lower color resistance to UV rays when contrasted with hair in the non-balding area. Hair color is mainly generated by melanin, and this experiment shows that POFs can effectively protect melanin from degradation by ultraviolet light.

### 2.5. Protein Loss Protection

The depletion of hair protein serves as a significant indicator of hair damage, with excessive protein loss leading to the curling of hair scales and various other hair-related issues. As depicted in Figure 2e, hair exhibited minimal protein loss in the absence of UV radiation. Conversely, balding hair subjected to 96 h of UV radiation without protection experienced a protein loss of 612.6 ± 30.24 μg/g, surpassing the protein loss observed in the occipital region of the scalp. Compared to the NC group, the POF group exhibited a protein loss of 421.5 ± 20.34 μg/g, while the VE group exhibited a protein loss of 490.13 ± 23.44 μg/g, demonstrating a protective effect on hair protein loss. Figure 2e reveals that the POF group displayed superior protection against hair protein loss in comparison to the VE group.

### 2.6. Tryptophan Protection

Amino acids play a significant role in maintaining hair health. UV radiation can lead to photodegradation, which disrupts the bonds between amino acids, resulting in the deterioration and loss of these essential components. This process ultimately contributes to the development of dry, fragile, and lackluster hair. Certain aromatic amino acids, such as tryptophan and tyrosine, are particularly susceptible to photodegradation. Despite its low concentration in keratin—only 0.7%—tryptophan is the amino acid that is most vulnerable to ultraviolet light exposure and subsequent photodegradation [24]. As such, it serves as a valuable indicator for assessing light-induced damage to hair. The tryptophan content in hair samples from the AGA hair loss area was determined using fluorescence, revealing a significant impact of UV radiation. Figure 2f indicates that the NC group’s fluorescence intensity (51.25 ± 3.67) of AGA balding hair decreased by 41.33% after 96 h of UV irradiation compared to 24 h of irradiation (intensity was 86.44 ± 5.67), while in the VE group, the intensity was 60.25 ± 4.81 after 96 h compared to 90.78 ± 6.63 after 24 h of UV irradiation, a subsequent 33.33% decrease in the luminous density value. In the POF group, the intensity was 69.12 ± 3.21 after 96 h and 92.53 ± 4.78 after 24 h, indicating that the fluorescence intensity of balding hair exhibited a reduction of 26.30% and suggesting that POFs may effectively mitigate the degradation of AGA hair caused by UV radiation. Furthermore, it was noted that the hair loss region affected by AGA was more vulnerable to UV-induced damage than permanent hair.

### 2.7. Tensile Properties

**Figure 3:**
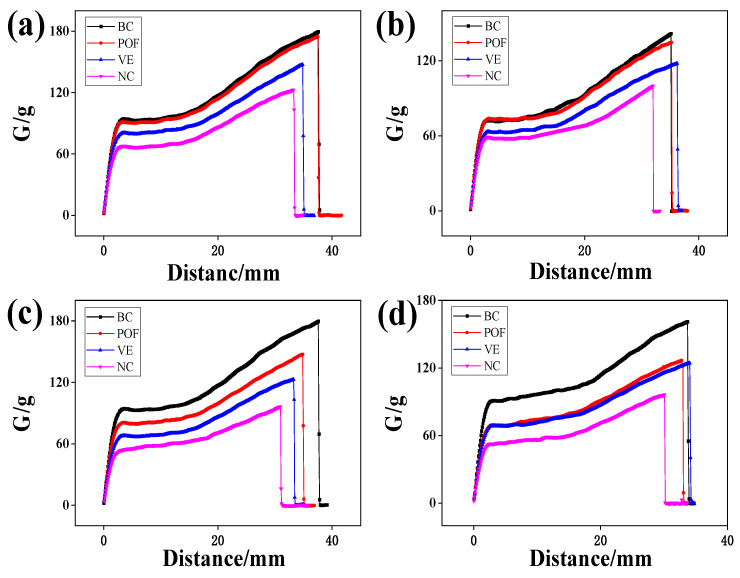
The effect of different irradiation times on the tensile properties of AGA balding hairs: (**a**) 24 h, (**b**) 48 h, (**c**) 72 h, (**d**) 96 h. Stress–strain curve of a single hair. To better reflect the proper stretching performance of the experimental hair, the average stretching performance of 50 hair samples was used in each experimental group.

Hair is considered the most elastic form of natural fiber, with its stretching capabilities contingent upon keratin’s presence in the cortical layer. This protein allows hair to stretch to up to twice its original length. Upon reaching a certain level of stretching, the α-helix structure within the hair is disrupted and transitions into a β-conformation, reducing elasticity [25]. A healthy hair cortex exhibits a balance of elasticity and toughness, whereas a damaged hair cortex displays diminished elasticity and fragility, making it prone to breakage. Hair elongation adheres to a specific pattern and aligns with the stress–strain curve, as illustrated in Figure 3. The elongation curve displays a point of inflection, progressing through three distinct stages: the pre-yield zone (the Hooke zone), the yield zone, and the post-yield zone. Within the pre-yield zone, a nearly linear correlation exists between alterations in stress and strain ratios. Keratin undergoes uniform elongation changes, preserving a spatially coiled state of binding forces among fiber macromolecules, such as hydrogen bonds and salt bonds. Subsequent elongation of the fibers causes them to pass through the first inflection point and enter the yield zone, during which protein fibers transform from α-helix to β-foldable structure. Upon further elongation, a second inflection point is reached, marking the entry into the post-yield region where disulfide bonds undergo deformation, ultimately resulting in the fracture of the hair. According to Figure 3, it is evident that POFs exhibit a notable ability to mitigate the detrimental effects of UV radiation on the performance of AGA balding hair lifting.

### 2.8. Preventing UV Damage of AGA Hair Using POFs

**Figure 4:**
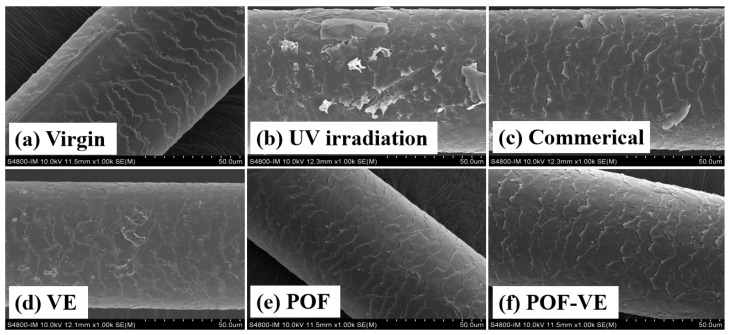
SEM images of AGA balding hair surface after being subjected to UV light for 72 h. Hair samples were treated with different protective formulations: (**a**) Negative control, (**b**) blank control, (**c**) Commercial products, (**d**) Vit E, (**e**) POFs, and (**f**) Vit E–POF.

AGA balding hair is susceptible to irreversible damage when exposed to UV radiation. Using a xenon lamp light source that replicates sunlight, hair was continuously irradiated for 72 h at wavelengths ranging from 300 to 800 nm and an amplitude of 300 W/m^2^. The resulting damage to the hair surface was significant, as depicted in Figure 4b. However, applying antioxidant substances to the hair, as shown in Figure 4e, led to a marked reduction in the damage caused by UV radiation. A comparative study was conducted to examine the efficacy of anti-UV hair care using POFs, in which antioxidant vitamin E, commercially available hair conditioners, and POFs were tested. Following 72 h of irradiation, the results depicted in Figure 4 indicate that POFs exhibited superior anti-UV effects compared to vitamin E and conditioners.

**Figure 5:**
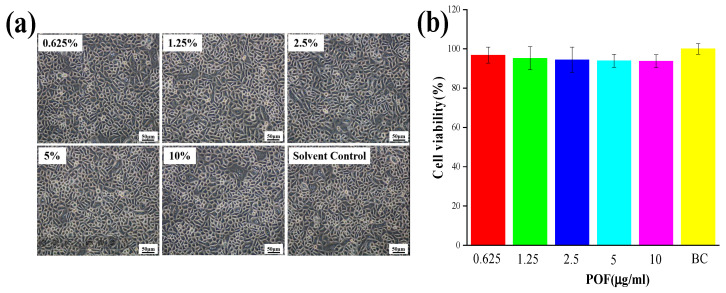
Cell viability testing of POFs. (**a**) Keratinocyte activity in response to POFs. (**b**) The effect of POF concentration on the activity of keratinocytes. Cell viability was analyzed using the MTT assay. BC (solvent control group): 200 μL culture medium. Data are presented as the mean ± SD (*n* = 3).

The stratum corneum of hair is established through the progressive keratinization of keratinocytes. To investigate the process through which POFs repair UV-induced hair damage, keratinocytes were utilized as experimental models for further investigation [26]. To evaluate the potential cytotoxic effects of POFs on keratinocytes, MTT assays were performed at varying concentrations over a 24-h period to ascertain the optimal dosage of POFs for subsequent experimentation. As depicted in Figure 5a, keratinocytes demonstrated increased proliferation in response to POF concentrations below 10 μg/mL. At this threshold, POFs did not induce notable toxicity in keratinocytes. Cell viability exceeding 90% was generally regarded as having a negligible influence on subsequent experimental outcomes. Noteworthy is the observation that keratinocytes exhibited a proliferation rate of 93.59% when exposed to a POF concentration of 5 μg/mL.

### 2.9. The Scavenging Ability of Free Radicals (ROS)

**Figure 6:**
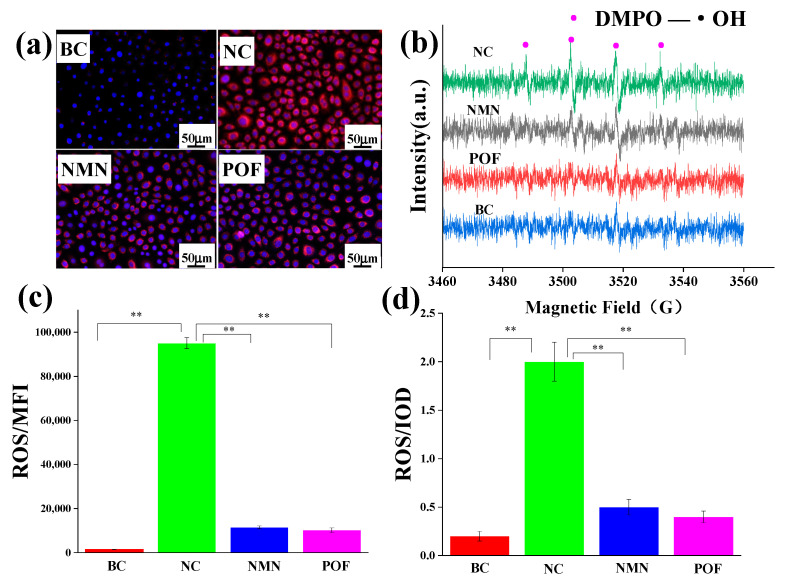
Testing the antioxidant capacity of POFs in keratinocytes. (**a**) Fluorescence images of mitochondrial ROS. (**b**) Image of hydroxyl radicals in keratinocytes captured by EPR under UV irradiation. (**c**) Test results of mitochondrial ROS. Integrated optical density (IOD) reflects the content of mitochondrial ROS. (**d**) Test results of ROS in keratinocytes. The average fluorescence intensity (MFI) reflects the content of ROS. BC: Keratinocytes were cultured without any treatment; NC: Keratinocytes were cultured without the addition of antioxidants but underwent ultraviolet irradiation; NMN: After culturing keratinocytes, NMN was added and subjected to ultraviolet irradiation; POF: After culturing keratinocytes, NMN was added and subjected to ultraviolet irradiation. The *t*-test was used for statistical analysis. A *p*-value < 0.01 is represented by **.

Free radicals have traditionally been recognized as a catalyst for oxidative stress. To assess the antioxidant potential of POFs, β-nicotinamide mononucleotide (NMN) was utilized as a positive control to evaluate its efficacy in scavenging free radicals. Following a 24-h incubation of keratinocytes, 2 mL of 2.5 μg/mL NMN and 2 mL of 2.5 μg/mL NMN POFs were added to the cell plate and cultured for 2 h. A fluorescent probe, DCFH-DA, was added and subsequently irradiated with 300 J/m^2^ UV light. ROS production by keratinocytes compared to the NC group without added antioxidants was analyzed using flow cytometry. Figure 6c reveals that, compared to the NC group lacking supplemental antioxidants, POFs demonstrated a notable capacity for ROS clearance.

Mitochondria serve as the primary sites of oxidation within the cell, making them the organelles with the highest levels of ROS activity. The DCFH-DA (2′,7′-Dichlorodihydrofluorescein diacetate) probe is a commonly used indicator of cellular ROS. It hydrolyzes into the fluorescent dye DCFH through an esterase reaction, which is then oxidized into a strong purple–red fluorescent product, DCF, in the presence of ROS, emitting a fluorescent signal. DCFH-DA can easily penetrate the cell membrane, allowing for the observation of sites with ROS within the cell using fluorescence microscopy. The ROS fluorescence microscope image presented in Figure 6a depicts the low levels of ROS within mitochondria in the absence of external interference, contrasting with the significantly elevated ROS content observed following exposure to UV radiation. Following POF treatment, the ROS levels were notably diminished post-UV irradiation compared to the NC group, as evidenced by the IOD values illustrated in Figure 6d.

ROS such as superoxide anion (O^2−^), hydrogen peroxide (H_2_O_2_), hydroxyl radical (•OH), ozone (O_3_), and singlet oxygen (^1^O_2_) [27] in keratinocytes were investigated using the electron paramagnetic resonance (EPR) technique to validate prior experimental findings [28]. Figure 6b reveals that keratinocytes did not capture free radicals without UV irradiation. In the absence of antioxidant substances, hydroxyl radicals were observed, using EPR spectroscopy, in the NC group under UV irradiation. Conversely, the introduction of NMN and POFs resulted in the absence of detectable free radicals under identical experimental conditions.

### 2.10. The Effect of UV Irradiation on Free Radicals in AGA Hair

**Figure 7:**
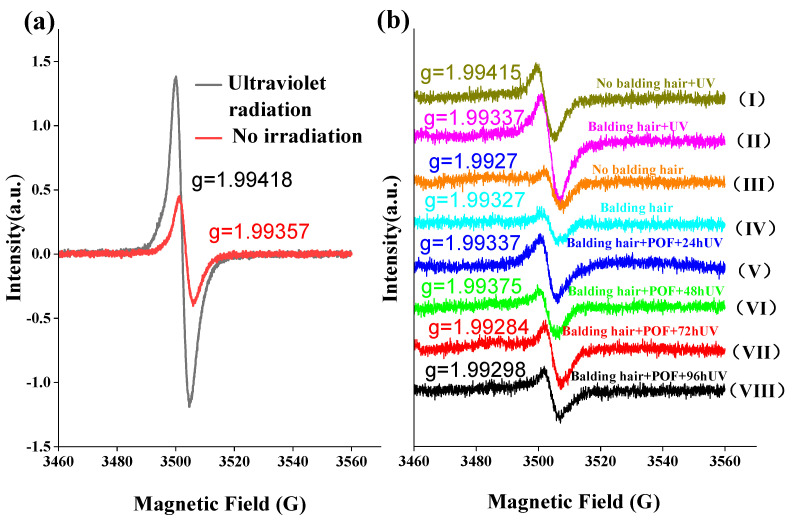
Testing free radicals in hair using EPR. (**a**) Comparison of hair in AGA balding areas under UV irradiation and non-irradiated free radicals. (**b**) Effect of POF treatment on hair free radicals in the AGA balding area. (I–IV): Comparison of free radicals in balding hairs and non-balding hairs before and after UV irradiation, (V–VIII) comparison of the free radicals generated by UV irradiation at different times after POF treatment of balding hairs.

Melanin, a uniform biopolymer with an intrinsic semiquinone radical, undergoes oxidation in the presence of free radicals upon exposure to sunlight, resulting in hair discoloration. The involvement of oxygen in the photoreaction of melanin is evident, as it is established that oxygen is utilized, and melanin free radicals are generated during the illumination process [29]. As depicted in Figure 7a, exposure to UV irradiation resulted in a notable increase in the expression of semiquinone free radicals in AGA hair compared to samples not subjected to UV irradiation. Furthermore, Figure 7b illustrates that the levels of semiquinone free radicals in the hair loss and non-hair loss areas of AGA were elevated under UV irradiation conditions, with no significant differences observed in the absence of UV irradiation. Furthermore, it is evident that semiquinone free radicals in AGA hair treated with POFs and subsequently exposed to UV irradiation decreased, with no discernible correlation to the duration of irradiation.

The electron paramagnetic resonance spectrum displays a singular isotropic signal with a g value ranging from approximately 1.993 to 1.994 and a line width of 5–6G, indicative of the presence of a semiquinone radical structure. Interestingly, the observed g value is lower than the expected g value of 2.0023 for a free electron. This discrepancy suggests a notable orbital coupling effect between the single electron and quinone within the system, resulting in substantial quenching of orbital angular momentum. This quenching offsets a portion of the spin angular momentum, ultimately leading to a g value below 2. The signal intensity serves as a measure of free radical concentration, with ultraviolet light irradiation resulting in a significantly higher signal compared to samples not exposed to irradiation. This difference is attributed to the photoelectric effect of ultraviolet light, which stimulates electron excitation and the formation of additional quinone free radicals. In the absence of UV light exposure, there is no notable disparity in free radical content between hair samples from areas experiencing hair loss and those from unaffected regions. When subjected to UV light irradiation, hair in the area affected by hair loss exhibits a greater abundance of free radicals compared to hair in unaffected areas, suggesting a faster degradation of hair in the former. Following the application of POF, a notable reduction in the concentration of free radicals in the affected hair region was observed. Despite prolonged exposure to 96 h of ultraviolet light, the levels of free radicals in the affected hair region either remained consistent or decreased below those found in unaffected hair regions, indicating the efficacy of POFs in inhibiting free radicals in hair.

## 3. Discussion

Recent studies have shown that plant extracts can help delay the photoaging of hair. In a study on hair repair using vegetable oil, it was found that grape seed oil has the highest glossiness, and based on color changes, safflower seed oil has the best glossiness effect [30]. All samples were subjected to tensile strain tests and rose fruit oil showed the best effect. Using scanning electron microscopy to observe changes in hair fractures, grape seed oil was the best for all hair types. In another study, it was found that Artichoke extract could enhance hair’s resistance to UV damage through antioxidant activity [31]. In an experiment on promoting hair growth with red ginseng oil, it was found that it can repair UV-damaged hair [32]. Meanwhile, in a study of liposomes for hair repair, it was found that vitamin E has a protective effect against UV-induced hair damage [20]. In a study on the repair of damaged hair using hair conditioners containing quercetin, it was found that quercetin has a good effect on repairing hair damage [33]. A common feature of the plant extracts studied is that they all have antioxidant properties. As shown in Table 1, it was found that flavonoids from *Platycladus orientalis* contain eight different flavonoid components, among which one type, quercetin, has been proven to have a repairing effect on hair damage. Antioxidant activity is an effective way of resisting UV damage, therefore, in this study, flavonoids from *Platycladus orientalis* were used to study resistance to UV damage to AGA hair. Vitamin E has been shown to repair hair damage and was used as a positive control in this study. Some studies have found that the growth cycle of immortal hair and forehead hair is different due to the influence of androgens [34]. This article aims to explore whether there are differences in the composition and force of the hair stem. Therefore, permanent hair from the back of the head of AGA patients was selected as the reference for the study.

The atrophy of hair follicles, thinning of hair shafts, and hair depigmentation primarily distinguish AGA. While researchers predominantly investigate the potential for hair regeneration in AGA, there is limited emphasis on hair quality in areas affected by hair loss. Exposure to sunlight can lead to photodegradation of human hair, with hair photoaging characterized by the lifting of hair cuticles [24], degradation of keratin [35], disruption of disulfide bonds [36], depletion of natural oils, loss of shine, heightened frictional resistance, and compromised mechanical properties [37]. Based on the findings presented in Figure 1b, it is evident that there was minimal hair damage in the balding region of AGA, characterized by raised scales, suggesting overall surface structural impairment. Following 72 h of UV irradiation, the SEM image in Figure 1d illustrates a more pronounced deterioration of the hair epidermis. Studies have demonstrated that the photooxidation of hair fibers involves the cleavage of C-S bonds in proteins, oxidation of internal lipids, oxidation of melanin particles, and degradation of tryptophan in keratin [38,39]. The predominant lipids in hair, primarily located on the hair surface, consist mainly of 18-MEA [40]. Consequently, upon exposure to UV radiation, these lipids are the initial targets for oxidation by light. Experiments have demonstrated that individuals with AGA exhibit the highest level of lipid peroxidation in their balding hair. The reduction in hair protein content suggests a weakening of the internal hair structure, leading to the shedding of melanin particles and a decrease in mechanical strength. Figure 2a reveals that the rate of protein loss in AGA-affected hair is accelerated when exposed to UV radiation. Furthermore, the shedding of melanin particles has implications for hair color, as melanin serves as a potent antioxidant that protects against light-induced damage. In its absence, hair becomes more susceptible to such damage.

UV radiation from sunlight has been found to contribute to the aging of the hair and scalp by causing disulfide bond breakage, oxidation of cysteine to alanine, degradation of tryptophan, and breakdown of melanin. Figure 6 indicates that exposure to UV radiation leads to the generating of numerous free radicals, specifically hydroxyl radicals, by keratinocytes. The interaction between highly reactive free radicals and intracellular structures results in cellular and tissue damage. The findings from the free radical scavenging experiment in keratinocytes suggest that POFs exhibit antioxidant properties, laying the groundwork for experiments on hair UV resistance. While the exact mechanisms of free radical production remain unclear, hydroxyl radicals (•OH) are commonly accepted as the main contributors to hair photodamage [17]. Experimental evidence also indicates the involvement of singlet oxygen (^1^O_2_) in photosensitization reactions [41,42]. Singlet oxygen (^1^O_2_) is a transient form of molecular oxygen that exhibits high reactivity towards unsaturated lipids [43], proteins [44], and nucleic acids [45], typically lasting only microseconds. The generation of singlet oxygen and its derivatives by melanin under UV irradiation has spurred extensive photophysical research [46]. This study further demonstrates that melanin free radicals are upregulated in response to light exposure to hair damage and pigment degradation. The addition of POFs can reduce this oxidative damage.

## 4. Materials and Methods

### 4.1. Materials and Reagents

*Platycladus orientalis* flavonoids (POF) were obtained from the research group’s laboratory. Hair samples were obtained from Chinese patients with AGA who were around the age of 38–42. The keratinocytes were purchased from Guangdong Bio Cell General Testing Co., Ltd., Guangzhou, China. Malondialdehyde (analytically pure), barbital thiosulfate (analytically pure), trichloroacetic acid (analytically pure), Coomassie Brilliant Blue G-250 (analytically pure), tryptophan (analytically pure), MTT, NMN, vitamin E, and DMSO were obtained from Sigma-Aldrich, St. Louis, MO, USA. Phosphate-buffered saline (PBS) and fetal bovine serum (FBS) were obtained from Beijing Solarbio Science & Technology Co., Ltd., Beijing, China. ROS reagent kits, Mito-Tracker Red CMXRos reagent kits, and DCFH-DA probes were purchased from Shanghai Beyotime Biotechnology Co., Ltd., Shanghai, China.

### 4.2. Extraction and Testing of POF Components

The *Platycladus orientalis* leaves were dried and then subjected to further drying in a 60 °C oven until they reached a constant weight. Subsequently, the dried leaves were ground using a grinder. A precise amount of 100 g of cypress powder was weighed and placed in a conical flask, followed by the addition of 95% ethanol at a ratio of 1:50. The mixture was then subjected to extraction for 20 min under ultrasonic power of 600 W, followed by extraction at a constant temperature of 30 °C for 6 h. The resulting solution was filtered, and the filtered residue was centrifuged. This extraction process was repeated twice, and the obtained filtrates were combined. The total flavonoid content in the filtrate was measured and the filtrate was concentrated using rotary evaporation at 45 °C to obtain the final POF powder. GC-MS (GCMS-QP2010 Plus, Shimadzu, Japan) was used to analyze the POF components, and the database was used to explore the flavonoids with higher content.

### 4.3. Preparation of Hair Samples and SEM Scanning

**Figure 8:**
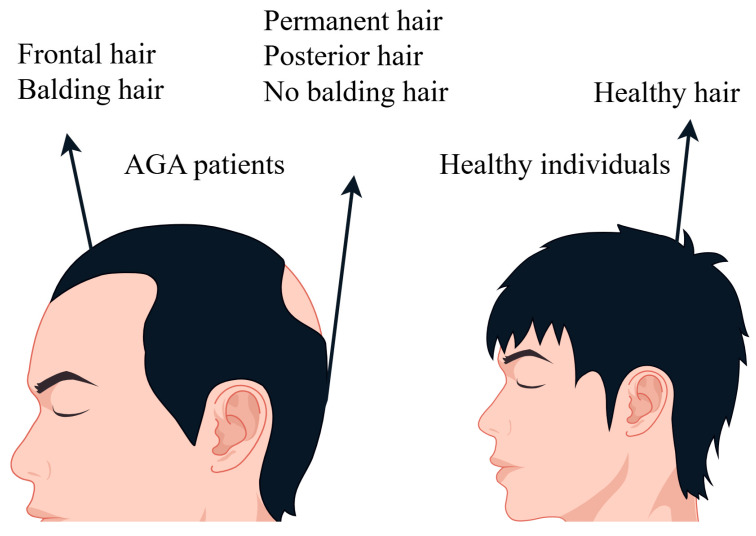
Hair samples from AGA patients and healthy individuals.

Hair samples were taken from 20 male AGA patients aged 38–42 who had been suffering from androgen hair loss for more than 5 years. Hair samples were taken from the frontal hair loss area (areas with sparse hair) and posterior head area, as shown in Figure 8, with 50 black hair samples taken from each area. The samples were cut into small pieces 10 centimeters long for later use. Healthy hair samples were obtained from 20-year-old non-AGA males. Cut the hair sample.

The samples underwent SEM using a cold field emission microscope (S-4800, Hitachi, Tokyo, Japan) following exposure to UV light. Each sample was securely attached to a metal stub using double-sided contact adhesive tape to maintain its integrity. Subsequently, gold was sputtered onto the samples under vacuum conditions. A comparative analysis was conducted between hair samples from individuals exhibiting balding, no balding, and healthy individuals.

### 4.4. UV Irradiation of Hair

To conduct the study more effectively, the hairs in this experiment were divided into several groups: sample groups (POF, POF-VE), positive control groups (VE, NMN), negative control group (NC), and blank control group (BC). Sample groups refer to the experiment conducted on hair after sample processing. The positive control group refers to hair treated with vitamin E, which has antioxidant properties. The negative control group refers to hair and cells that have been directly irradiated with ultraviolet radiation without adding any samples. The blank control group refers to hair or cells that have not undergone any treatment.

The hair samples were initially washed with non-ionic shampoo and allowed to dry. Subsequently, they were treated with POFs at a concentration of 5% for 30 min, followed by exposure to UV irradiation. Hair samples not exposed to UV were designated as the control group, while those treated with vitamin E were the positive control group. This experimental procedure was repeated over a period of four days. Subsequently, light-damaged hair samples were washed with non-ionic shampoo and air-dried before further analysis. UV irradiation was carried out using a UV radiation source (Shenzhen Anhongda Optoelectronic Technology Co., Ltd., Shenzhen, China) at a dose of 300 J/m^2^ [47].

### 4.5. Lipid Peroxide Test

Standard malondialdehyde solutions (0, 0.2, 0.4, 0.6, 0.8, and 1.0 mL of 100 μg/mL concentration) were dispensed into six 10 mL volumetric flasks. A 5 mL volume of 7.5% (*w*/*v*) trichloroacetic acid standard solution and 4 mL of 0.5% (*w*/*v*) barbital thiosulfate standard solution were added to each flask and then diluted with distilled water. The solutions were thoroughly mixed and then allowed to equilibrate to generate a series of standard solutions. The absorbance of each solution was determined at a wavelength of 520 nm using a UV spectrophotometer (T2602S, Shanghai Youke Instrument and Meter Co., Ltd., Shanghai, China), and then a standard curve was generated.

A total of 500 mg of AGA hair was taken and cut into small pieces measuring approximately 0.5 cm in length. Subsequently, 10 mL of methanol was added to the hair samples and subjected to sonication for 10 min. Then, 2 mL of the resulting upper layer solution was transferred into a small beaker and 10 mL of a 7.5% trichloroacetic acid solution was introduced into the beaker and the mixture agitated for 30 min. Upon completion, the solution was filtered, and 5 mL of the filtrate was extracted and combined with 5 mL of a 0.5% (*w*/*v*) barbital thiosulfate standard solution. Subsequently, the solution was immersed in a water bath set at 90 °C for 40 min, followed by immediate cooling to yield the test solution. Finally, the absorbance was measured at 520 nm using a UV spectrophotometer [48].

### 4.6. Chromatism

The color changes of a standardized bundle of hair were assessed using a colorimeter (Datacolor-600, Lawrenceville, NJ, USA) at designated points prior to UV irradiation and at 24-, 48-, 72-, and 96-h post-irradiation. The degree of hair damage and the efficacy of various samples in protecting the hair were evaluated using the ΔE metric. Three measurements were taken at each test point and averaged for analysis [49].

### 4.7. Protein Loss

Standard protein solutions (0, 0.2, 0.4, 0.6, 0.8, and 1.0 mL of a 1 mg/mL concentration) were dispensed, in triplicate, into individual 10 mL test tubes. Each sample was diluted with deionized water to a final volume of 1 mL. A 1 mL volume of each diluted sample was combined with 5 mL of Coomassie Brilliant Blue G-250 solution in a fresh test tube. The contents were thoroughly mixed and allowed to incubate for 2 min. The absorbance of the solution was determined at a wavelength of 595 nm using a UV spectrophotometer, and a standard curve was then generated.

The hair samples were cut into small pieces approximately 0.5 cm long and placed in a 25 mL conical flask with a stopper. A 1.5 g hair sample was then mixed with 10 mL of distilled water in the flask, which was stopped and shaken for 4 h at room temperature using a desktop constant temperature shaker set at 150 rpm. Following this, 1 mL of the turbid liquid suspended on the surface was transferred using a pipette, 5 mL of Coomassie Brilliant Blue G-250 dye solution was added, and the volume was adjusted to 10 mL. The absorbance at 595 nm wavelength was measured using a UV spectrophotometer, and then the protein loss of the samples was calculated based on the standard curve equation [50].

### 4.8. Tryptophan Content Test

The AGA hairs were meticulously cleaned and evenly distributed before being subjected to irradiation under a UV lamp. Samples were then collected at predetermined intervals, and the tryptophan fluorescence intensity was measured using a fluorescence spectrophotometer (FL1009M020 VARIAN, Agilent, Santa Clara, CA, USA). Hair that had not been exposed to UV irradiation served as the blank control (BC) group. Hair (20 mg) was cut into small pieces measuring approximately 0.5 cm. Subsequently, 4 mL of a 5.5 mol/L sodium hydroxide solution (referred to as tryptophan hydrolysis solution) was added. A nitrogen blower was used to pass nitrogen gas through the mixture, sealed, and placed in a 110 °C oven for hydrolysis over 20 h. Once completed, the mixture was allowed to cool in a dark environment until it reached room temperature. The solution was transferred to a 25 mL volumetric flask (known as a colorimetric tube) and the pH adjusted to 5–6 using 0.1 mol/L of hydrochloric acid. The pH was further adjusted to 7–8 using 0.5 mol/L of sodium oxide. The flask was filled with distilled water, ensuring thorough mixing, and then the solution was centrifuged at a speed of 3000 rpm for 30 min. Using a fluorescence spectrophotometer with excitation at 290 nm and emission at 360 nm, the relative fluorescence intensity of the test solution was measured to analyze the tryptophan content in hair. Amino acids are susceptible to photodegradation and loss when exposed to UV irradiation, leading to a reduction in content. A decrease in the extracted amino acid content in hair indicates greater photodamage to the hair [51].

### 4.9. Mechanical Properties of Hair

The tensile strength of hair was evaluated using the Brookfield Ametek CTX (Brookfield, MA, USA) instrument at an ambient temperature of 25 °C, with a configuration consisting of a 5 KG force sensing element, 0.1 g resolution, and TA-DGF001 stretching fixture. The TA-DGF001 stretching fixture was installed and adjusted to a specified distance of 140 mm for this experiment. The accuracy of the test results is directly influenced by the effective measurement of the length of the hair, necessitating consistent hair positioning for each test. A single hair thread was carefully placed and secured on the fixture, with attention given to the relaxation of the hair thread. The initial reading of the sensor was calibrated to zero before the experiment. The testing parameters were established to be a stretching trigger value of 1 g, a testing speed of 1 mm/s, and a stretching distance of 50 mm. Data for the distance and breaking force values of the hair wire breakage were recorded [52].

### 4.10. Antioxidant Test

Keratinocytes were seeded into a 6-well plate and incubated overnight in a CO_2_ incubator (37 °C, 5% CO_2_) following resuscitation. A 2 mL volume of sample was added to each well, with 3 wells allocated to each experimental group (blank, POF, negative, and positive NMN). The blank control and negative groups received 2 mL of culture medium. Subsequently, the plates were placed in a CO_2_ incubator (37 °C, 5% CO_2_) for 24 h. UVB irradiation at a dose of 300 mJ/cm^2^ was applied to all groups except the blank control before further analysis.

The Keratinocytes ROS test involved irradiating cells in each well, followed by three washes with PBS and the addition of 1 mL of a 10 μM DCFH-DA probe. The cells were then incubated in a CO_2_ incubator at 37 °C with 5% CO_2_ for 30 min. Subsequently, the culture medium containing DCFH-DA was removed, and the keratinocytes were washed three times with PBS, digested with trypsin (0.25%), and washed again with PBS before being subjected to flow cytometry cell analysis [53].

The mitochondrial ROS test involved treating keratinocytes with UV radiation, fixing them with 4% paraformaldehyde for 30 min, washing them with PBS for 8 min, wiping the surrounding liquid, and sealing them with a fluorescence anti-quenching sealing tablet. The samples were then observed under a microscope (Leica, DM2500, Wetzlar, Germany) and analyzed using Image-Pro^®^Plus 6.0 image processing software. The ROS removal rate was calculated using a specific formula.

$$\mathrm{ROS}\;\mathrm{removal}\;\mathrm{rate}\;(\%)=\frac{OD\left(NC\right)-OD(SC)}{OD(NC)}\times 100\%.$$

### 4.11. EPR

Electron paramagnetic resonance (EPR) (Bruker Instruments, Saarbrücken, Germany) measurements were conducted to characterize specific ROS. Cells were treated with 5,5-dimethyl-1-pyrroline-N-oxide (DMPO) at a concentration of 10 mM for 10 min at 37 °C, in the presence or absence of ROS modulators. EPR signals were recorded using a Bruker EMX spectrometer (Billerica, MA, USA) with a flat cell assembly. Hyperfine couplings were determined to an accuracy of 0.1 G by measuring the magnetic field separation, with potassium tetraperoxochromate and 1,1-diphenyl-2-picrylhydrazyl as reference standards. Data were acquired and analyzed using a dedicated software program (Bruker Instruments) [54].

### 4.12. Statistical Analysis

Data visualization and analysis were conducted using GraphPad Prism software 9. The results are presented as Mean ± SD. Statistical comparisons between groups were performed using two-tailed *t*-tests. A significance level of *p* < 0.05 was considered statistically significant.

## 5. Conclusions

This research explores the impact of flavonoids derived from *Platycladus orientalis* leaves on the restoration of hair damaged by AGA. A comparative analysis was carried out on AGA-affected hair treated with POFs before and after UV radiation exposure. The findings suggest that POFs can mitigate the detrimental effects of UV radiation on AGA-damaged hair. The study also examined the depletion of protein and lipid peroxides from hair, alterations in hair color, loss of tryptophan from hair, and modifications in hair mechanical properties. Following POF treatment, a notable decrease of 59.72% in peroxidized lipids was observed in AGA hair, resulting in a deceleration of the hair color change. Additionally, protein loss was reduced by 191.1 μ/g, and tryptophan loss by 15.03%, thereby improving the hair’s tensile strength. It is worth noting that under UV irradiation, the AGA hair exhibited more pronounced losses of protein, color differentiation, lipid peroxidation, and tryptophan compared to permanent hair. Studies investigating the protective mechanism of POFs have demonstrated that they shield AGA-affected hair from UV-induced damage by scavenging hydroxyl radicals with antioxidants and reducing melanin free radicals generated by UV radiation.

## Figures and Tables

**Table 1:** Analysis of flavonoids in *Platycladus orientalis*.

Peak Time (min)	Component	Content (%)	CAS
3.824	Myricetin	5.15	529-44-2
4.490	Quercetin	1.34	522-12-3
5.113	Afzelin	1.54	482-39-3
5.770	Aromadendrin	2.28	480-20-6
6.277	Hinokiflavone	4.83	19202-36-9
7.061	Amentoflavone	4.37	1617-53-4
10.226	Kaempferol	2.41	520-18-3
13.342	Rutin	2.35	153-18-4

## Data Availability

The data presented in this study are available on request from the corresponding author. The data are not publicly available due to specific ethical and privacy considerations.
